# P-319. Genetic shift of Methicillin-resistant *Staphylococcus aureus* isolated from bloodstream infections over 12 years at a tertiary care university hospital in Japan

**DOI:** 10.1093/ofid/ofae631.522

**Published:** 2025-01-29

**Authors:** Yuka Motomura, Motoyasu Miyazaki, Mitsuhiro Kamada, Tomomitsu Satho, Tohru Takata, Nobuhiro Kashige

**Affiliations:** Fukuoka University, Fukuoka, Fukuoka, Japan; Fukuoka University, Fukuoka, Fukuoka, Japan; Fukuoka Unviersity, Fukuoka, Fukuoka, Japan; Fukuoka University, Fukuoka, Fukuoka, Japan; Fukuoka University, Fukuoka, Fukuoka, Japan; Fukuoka University, Fukuoka, Fukuoka, Japan

## Abstract

**Background:**

Limited reports exist on the long-term trends in genotypes of Methicillin-resistant *Staphylococcus aureus* (MRSA) bloodstream isolates. Therefore, we aimed to investigate the longitudinal trends in genotypes of MRSA bloodstream isolates obtained from hospitalized patients during a 12-year study period from 2010 to 2021 at a tertiary care university hospital.

**Methods:**

Over the 12-year period spanning from 2010 to 2021, we conducted a genetic investigation focusing on MRSA 245 strains isolated from the blood of hospitalized patients. The analysis involved the application of Staphylococcal Cassette Chromosome *mec* (SCC*mec*) typing, accessory gene regulator (*agr*) typing, PCR-based ORF Typing (POT), and Multi Locus Sequence Typing (MLST) to discern the genotypes of the MRSA bloodstream isolates. In this study, strains with the same POT type detected in two or more isolates were designated as epidemic clones, while strains without a common POT type were classified as sporadic clones.

**Results:**

Until 2015, isolates with SCC*mec* II/*agr* II were prevalent, but from 2016 onwards, there was an increase in isolates with SCC*mec* IV/*agr* III. A total of 128 strains (52%) were identified as epidemic clone, while 117 strains (48%) were classified as sporadic clone. The detection rate of sporadic clone had significantly increased since 2016 (P < 0.05). The epidemic clone was classified into three clusters, with MRSA of CC1 being prominently detected after 2016.

**Conclusion:**

This study found that the genotypes of MRSA bloodstream isolates underwent a shift from SCC*mec* II/*agr* II type to SCC*mec* IV/*agr* III type, particularly with a notable increase in MRSA of CC1, after the year 2016. There was a significant increase in the proportion of sporadic strains among the isolates, suggesting a diversification of genotypes.

**Disclosures:**

**All Authors**: No reported disclosures

